# Professionalism in nursing in Ethiopia: systematic review and meta-analysis

**DOI:** 10.3389/fmed.2025.1549109

**Published:** 2025-04-23

**Authors:** Afework Edmealem, Temesgen Ayenew, Setarg Ayenew, Belachew Tegegne, Sewunet Ademe, Dereje Esubalew, Addisu Getie, Tiliksew Liknaw

**Affiliations:** ^1^Department of Nursing, College of Medicine and Health Science, Debre Markos University, Debre Markos, Ethiopia; ^2^Department of Nursing, College of Medicine and Health Science, Injibara University, Injibara, Ethiopia; ^3^Tropical College of Medicine, Dessie, Ethiopia

**Keywords:** nursing, nurses, Ethiopia, caring behavior, factors, professionalism in nursing

## Abstract

**Background:**

In any field, professionalism is essential. A profession can fulfill its responsibilities when professionals develop their knowledge in various ways, such as continuing professional development, expanding their skill levels, adhering to the norms of the profession, and demonstrating high levels of commitment. Developing professionalism in nurses is a key strategy for improving the quality of nursing care and healthcare. However, no study has shown a comprehensive overview of professionalism in nursing in Ethiopia. Thus, this systematic review and meta-analysis aim to present a comprehensive assessment of the overall level of professionalism in nursing in Ethiopia.

**Methods:**

The Preferred Reporting Items for Systematic Review and Meta-Analysis standard was followed in the reporting of this systematic review and meta-analysis. An extensive exploration of digital repositories, including PubMed (MEDLINE), EMBASE, Cochrane, Africa Journal of Online, Google Scholar, and an advanced Google search, was conducted to obtain published studies detailing professionalism in nursing in Ethiopia. STATA version 17 commands created the pooled estimate with a 95% confidence interval. The I^2^ test and Egger’s test were used to identify the presence of heterogeneity and publication bias, respectively. To manage heterogeneity, a subgroup analysis and random effect model were used.

**Results:**

A total of 11 articles with a total of 3,581 participants were included in the final systematic review and meta-analysis. The pooled estimate of professionalism in nursing in Ethiopia was 54% (95% CI: 44, 66%). In the subgroup analysis, the highest pooled estimate of professionalism in nursing was observed in South Ethiopia, which is 64% (95% CI: 43–86%).

**Conclusion:**

The level of professionalism in nursing in Ethiopia is suboptimal. Being female, having a higher educational level, having long years of experience, having a low workload, having favorable job satisfaction, being a member of a nursing organization, having a good working environment, working in non-stressful units, and having a good organizational culture were the major factors that had a positive association with professionalism in nursing. Therefore, healthcare professionals, the Ministry of Health, and other stakeholders should focus on interventions to enhance the organizational culture, job satisfaction, working unit, and working schedule for nurses.

## Introduction

The term ‘professionalism’ is broad and has various meanings. It can be characterized as possessing the necessary information and abilities to carry out tasks and roles effectively, communicate clearly, and act morally in all circumstances (1). According to other definitions, it is the collection of mindsets and ways of acting considered suitable for a specific line of work (2, 3). The American Nurses Association (ANA) lists six characteristics of nursing professionalism. These include giving care, establishing a relationship, combining expertise and objective data, using the body of knowledge to diagnose and treat human reactions, expanding nursing knowledge via research, and impacting policy (4). A profession can fulfill its obligations when its members demonstrate a high degree of dedication, grow their skill set, and use diverse methods to enhance their knowledge (3).

In nursing, professionalism is a fundamental and crucial idea that benefits both communities and patient care. Consequently, in the past few decades, nursing professionalism has gained international attention (2, 3). Despite this, the nature and emotional demands make it difficult for nurses to be true professionals. More intriguingly, the changing healthcare system, increasing societal, financial, and professional demands on the next generation of nurses, along with inadequate institutional responsiveness in both service and education, contribute to the growing concern about nursing professionalism (5). In order to become actual professionals, nurses should exhibit their profession to the public and relevant bodies through practicing (6).

In the current century, healthcare systems focus on providing quality healthcare service, satisfying customers, achieving optimal patient outcomes, improving societal perceptions, achieving health-related indicators, fostering effective collaborative practice among teams, and ensuring job satisfaction among the healthcare workforce (6). To achieve these health indicators and maintain quality healthcare service, professionalism in nursing is highly required, and it is critical to make a detailed assessment of both the entirety of the profession and the individual behaviors that comprise professionalism (7).

Providing basic professional nursing care has been reported to result in improved patient satisfaction and positive health outcomes (8). A high level of professionalism in nursing is associated with outcomes of improved nursing performance, enhanced critical thinking, the ability to reflect on practice, and increased empowerment. In addition, nurses with a higher level of professionalism have been reported to have increased job satisfaction (9). On the contrary, a lack of professionalism minimizes public expectations, as the public assumes the profession should prioritize their interests. As a result, acceptance of the profession becomes low, and its reputation suffers. This can also lead to a loss of standards and self-regulation, contributing to high turnover, burnout, fatigue, and reduced productivity (8, 10).

As a profession, nursing faces challenges such as lack of autonomy and leadership skills, healthcare risks, lack of leadership, long working hours, lack of recognition from the public, burnout, fatigue, high intention to leave the profession, and emotional load (3). Such challenges are a major obstacle to the growth and development of the profession (11). The new generation is choosing professions that are valued by society and by professionals themselves (12). Currently, nursing has become less attractive, with fewer individuals choosing the profession due to stressful working conditions, disrespect from the public, low salaries, and lack of prestige (6). Because of this, now is the right time to explore professionalism and implement corrective actions to expand and maintain the profession at the national level. Thus, this systematic review and meta-analysis were carried out to assess the pooled estimate of professionalism in nursing in Ethiopia.

## Aim of the study

Overall, the aim of this systematic review and meta-analysis is to provide a comprehensive understanding of the current state of nursing professionalism in the country, identify areas for improvement, and offer insights into informed policymaking, education, and practice within the nursing profession.

## Methods

### Study design and setting

A systematic review and meta-analysis of published articles in Ethiopia were conducted to estimate the level of professionalism in nursing among nurses in Ethiopia. Ethiopia is one of the developing countries in the eastern part of Africa. It has 13 regions and two city administrations. The regions are Tigray, Afar, Amhara, Oromo, Ethiosomali, Benishangul-Gumuz, Central Ethiopia, Sidama, South West Ethiopia, South Ethiopia, Gambela, and Hareri. The two city administrations are Addis Ababa and Dire Dawa (13).

### Eligibility criteria

#### Inclusion criteria

In this systematic review and meta-analysis, all published articles in Ethiopia were included. All studies that reported on the level of professionalism in nursing or the caring behavior of nurses were included. All types of studies published in English and conducted in all specialties of the nursing profession and those published from 10 January 2004 until 10 January 2024 were included.

#### Exclusion criteria

Studies that failed to report professionalism in nursing were excluded from this review. Additionally, studies such as case reports, case studies, editorial letters, and reports were excluded from the study.

#### Data source and search strategy

Before going through the extensive search, a systematic review and meta-analysis protocol on professionalism in nursing and caring behavior at PROSPERO was checked. After that, all published articles that reported the level of professionalism in nursing or the caring behavior of nurses in all regions of Ethiopia were used as data sources. The review was conducted using the Preferred Reporting Items for Systematic Review and Meta-Analysis (PRISMA) guidelines (14). The search strategy was developed using the Population Intervention Comparison and Outcome (PICO) search guide. An intensive search of online databases such as PubMed (MEDLINE), EMBASE, Cochrane, Africa Journal of Online, Google Scholar, and an advanced Google search was carried out. During a systematic search in the PubMed engine, the MeSH words “professionalism” OR “caring behavior” AND nursing OR “nurses” AND “factors” were used. All articles in the reference lists were searched to include additional studies and reports in the review and analysis. A systematic search of the literature was carried out from 1 January 2024 to 10 January 2024.

#### Measure of outcome

Professionalism in nursing refers to the actions, conduct, and mannerisms exhibited by professional nurses that convey concern for patients, ensure safety, provide attention, and uphold the integrity of the profession.

#### Quality assessment and critical appraisal

In this systematic review and meta-analysis, the included studies were cross-sectional studies. Therefore, the quality of included studies was assessed by an 8-item critical appraisal tool for cross-sectional studies adopted from the Joanna Briggs Institute (JBI) (15) and was considered high quality if the JBI score was above 70. This is a tool used for the evaluation of prevalence studies. **AE** and **TL** assessed the methodological quality of eligible articles independently. The differences in extraction were managed through discussion. All articles that scored above half of the score were included in the systematic review and meta-analysis.

#### Data extraction/abstraction

A Microsoft Excel spreadsheet was used to generate the pre-piloted format in which AE and SA extracted the data from the included literature. First author names, year of publication, region, sample size, study design, prevalence of professionalism, and factors associated with professionalism were retrieved from each article. Following a conversation among the authors to resolve any disagreement, AE collated the data extracted from the authors.

#### Statistical analysis

The extracted data were exported from the Microsoft Excel spreadsheet and entered into the STATA version 17 command window. Using the I-squared statistic, the presence of statistical heterogeneity within the included papers was evaluated before conducting the primary meta-analysis. I-squared values in the approximate range of 0–40%, 30–60%, 50–90%, and 75–100% may indicate low, moderate, substantial, and considerable heterogeneity, respectively. Additionally, Egger’s tests and a funnel plot were used to determine whether publication bias existed. Following that, the STATA meta set command was used to complete the pooled estimate. To control heterogeneity, a subgroup analysis of the included studies was conducted based on the categories of populations and the cutoff point. Additional advanced statistical analyses, such as meta-regression to identify the potential sources of heterogeneity and sensitivity analysis to investigate the influence of a single study on the overall pooled estimate, were performed. The trim and fill tests were conducted to minimize publication bias. The findings of this study were presented using tables and forest plots with a 95% confidence interval (CI).

## Results

### Identification of studies

**Figure 1:**
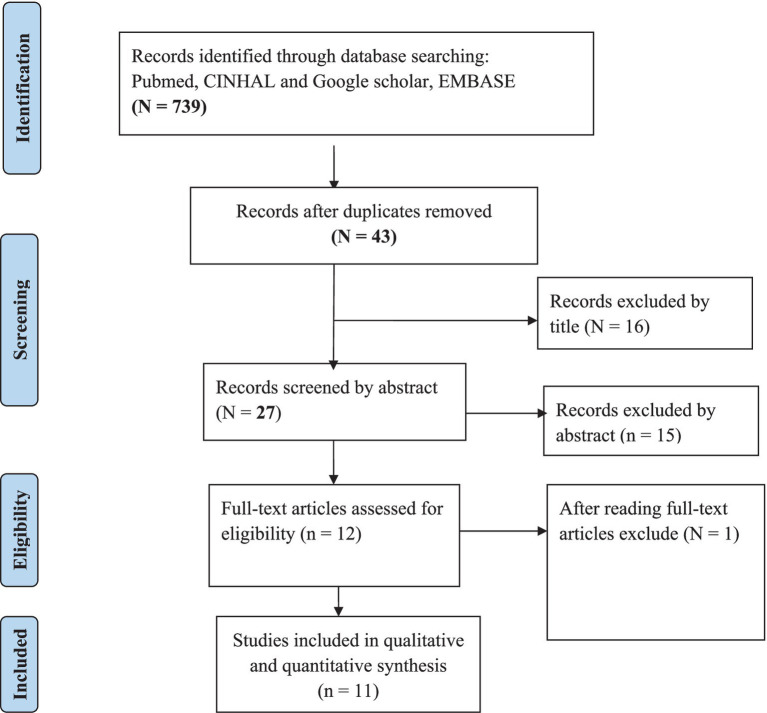
PRISMA flow diagram shows study selection for meta-analysis professionalism in nursing in Ethiopia, 2024.

Using advanced searching, a total of 739 articles were found in PubMed (MEDLINE), EMBASE, Cochrane, Africa Journal of Online, and Google Scholar. Reference tracing was used to add articles to the overall number of articles. A total of 43 articles were left after removing duplicates. A total of 27 articles were screened using their abstract after removing 16 articles by their title. Following that, a total of 12 articles were screened by reading their full text, and finally, a total of 11 articles passed the eligibility requirements, and quality assessment was included in the final systematic review and meta-analysis (Figure 1).

### Description of the included studies

A total of 11 articles with 3,581 study participants were included in the final systematic review and meta-analysis. All the searched articles were published from 2014 to 2023. Of the total included studies, two of them were conducted in the Amhara region (11, 16), and four studies were conducted in the Oromia region (7, 17–19). The other two articles were conducted in South Ethiopia (20, 21), and the two articles were conducted in the Hareri region (22, 23). The remaining article was conducted in the Tigray region (24). Regarding sampling technique, two articles used simple random sampling to select their participants (17, 23), and five articles used systematic sampling (7, 11, 16, 20, 21). The other four articles used a non-probability sampling technique (18, 19, 22, 24). The largest sample size was 465, which was obtained from a study conducted in the Hareri region in 2022 (23) (Table 1).

**Table 1:** Characteristics of included studies for professionalism in nursing in Ethiopia, 2024.

Author	Publication year	Region	Sampling technique	Sample size	Professionalism in nursing (%)	Factors associated with professionalism in nursing
Abate et al. (11)	2021	Amhara	Systematic Sampling	407	24.8	Experience > 10 years, working on the day shift, being a member of the organization, having life insurance
Gizaw et al. (18)	2018	Oromia	Census	321	36	Holding a position, organizational commitment, job satisfaction
Ashagere et al. (21)	2023	South Ethiopia	Systematic sampling	360	53.3	Being married, work experience (0–5, 6–10), satisfaction with motivation and prospect, and satisfaction with the nursing
Fikre et al. (23)	2022	Hareri	Simple random sampling	465	63.4	Degree and above, low workload, being satisfied with the job, having collaboration with others
Kibret et al. (22)	2022	Hareri	Census	300	51.6	Good working environment, favorable job satisfaction, low workload
Tola et al. (12)	2020	Oromia	Census	380	58.7	Being female, job satisfaction, having training
Oluma et al. (17)	2020	Oromia	Systematic	224	80	Personal satisfaction, professional job satisfaction, working environment (satisfaction by nurse managers, satisfaction by nursing staff and support negatively), collaboration with nurses and physicians, working unit (surgical ward negatively and ICU negatively)
Solomon et al. (7)	2015	Oromia	Systematic sampling	303	30.3	Organizational culture, being female, self-image, being single, high level of education, salary
Fantahun et al. (24)	2014	Tigray	Convenient	210		Longer years of experience, old age, being a member of the organization, diploma nurses, working in military hospitals, having a moderate attitude toward the nursing profession, lack of health insurance, less attention from nursing associations, and workload
Bekalu et al. (16)	2023	Amhara	Systematic	350	68.6	Being female, having association membership, having a positive self-image, having a good organizational culture, and having job satisfaction
Assefa et al. (20)	2022	South Ethiopia	Systematic	261	75.1	Old age, job satisfaction, cooperation, and a low workload

### Heterogeneity test and publication bias

**Figure 2:**
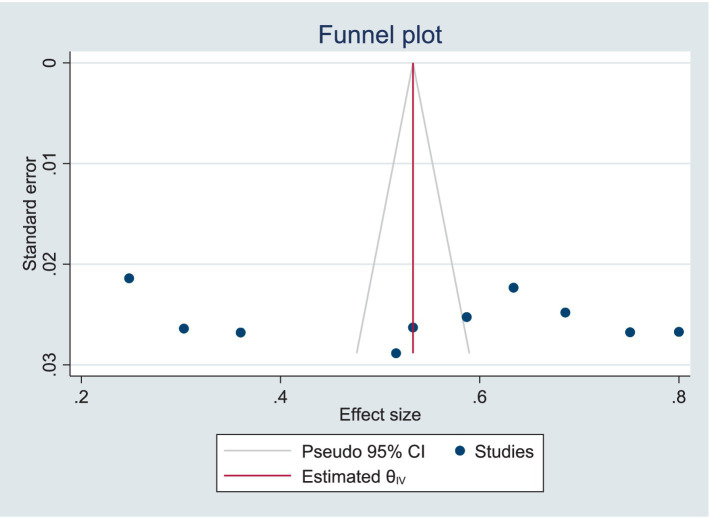
Funnel plot to see the presence of publication bias, 2024.

Before pooling the estimated effect, the presence of heterogeneity and publication bias was evaluated. The heterogeneity between the included articles was assessed statistically using the I-squared statistic. According to the results, there was high heterogeneity between the included studies (I^2^ = 98.19; *p* = 0.00). The presence of publication bias was statistically assessed using Egger’s weighted regression and the funnel plot. The *p*-value of Egger’s and Begg’s test was 0.5239, which indicates that there is no publication bias. However, the funnel plot showed the asymmetrical distribution of studies inside the funnel, which implies that there is a publication bias (Figure 2).

### Professionalism in nursing in Ethiopia

**Figure 3:**
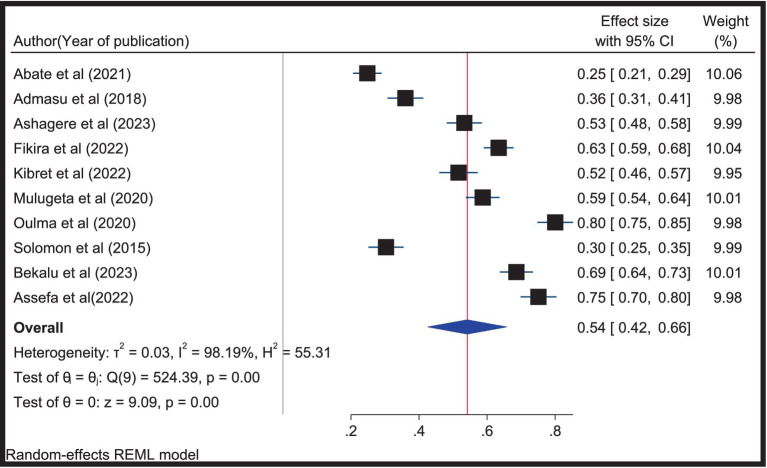
Forest plot depicting the pooled prevalence of professionalism in nursing in Ethiopia, 2024.

After testing for heterogeneity and publication bias, the effect size was pooled using STATA version 17 from 10 included studies. The remaining article was excluded from the analysis as it did not report the overall professionalism, although it was included when extracting factors. Since there was considerable heterogeneity between the studies (I^2^ = 98.19; *p* = 0.00), the main meta-analysis was performed using a random effect model. As shown in Figure 3, the pooled estimate of professionalism in nursing in Ethiopia was 54% (95% CI: 42–66%) (Figure 3).

### Meta-regression

Meta-regression was performed to identify the sources of heterogeneity between the studies. In this systematic review and meta-analysis, meta-regression was performed using the region and sampling technique to evaluate whether these variables are sources of heterogeneity between the studies. However, none of them were sources of heterogeneity, in which the *p*-value was above 0.05 (Table 2).

**Table 2:** Meta-regression of selected variables for professionalism in nursing in Ethiopia, 2024.

Heterogeneity sources	Coefficients	Std. error	*P*-value
Region	0.017	0.047	0.364
Sampling technique	−0.130	0.067	0.151

### Subgroup analysis for professionalism in nursing

**Figure 4:**
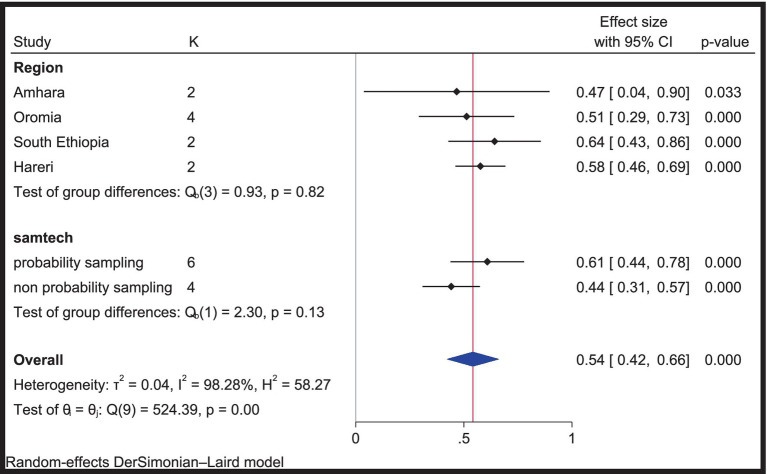
Subgroup analysis to minimize heterogeneity of included studies, 2024.

Subgroup analysis was performed using region and sampling techniques to minimize heterogeneity between the included studies. As shown in Figure 4, the highest pooled estimate of professionalism was observed in studies conducted in South Ethiopia, at 64% (95% CI: 43–86%). The pooled estimate of professionalism in studies that used probability sampling (simple random sampling and systematic sampling) was 61% (95% CI: 44–78%). However, there is no difference between the pooled estimate of each subgroup and the overall pooled estimate. All the pooled estimates of professionalism in each subgroup were between 42 and 66% (Figure 4).

### Sensitivity analysis

**Figure 5:**
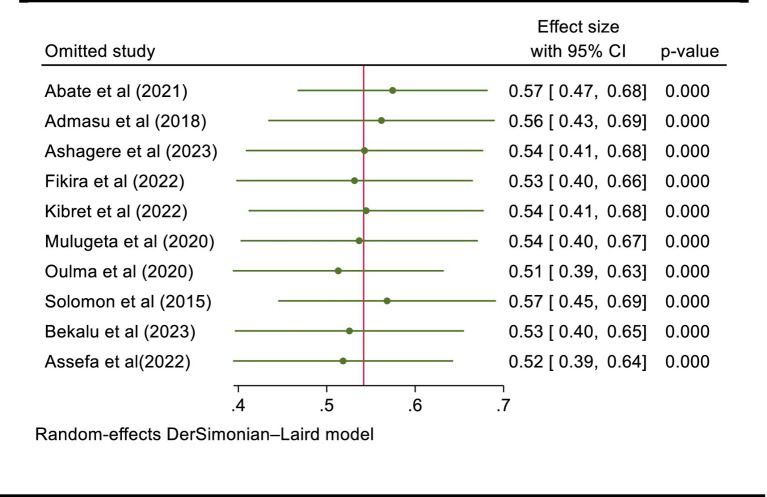
Leave-one-out meta-analysis to observe the influence of each study on the pooled effect size of professionalism in nursing, 2024.

Sensitivity analysis was performed to evaluate whether the pooled effect size was influenced by individual studies. As shown in Figure 5, there is no study that influences the overall pooled professionalism in nursing. The pooled effect size after omitting the individual study was within the confidence interval of the overall pooled effect size (all the effect sizes after omitting a single study were between 42 and 66%) (Figure 5).

### Factors associated with professionalism in nursing

In this systematic review and meta-analysis, different factors that had a significant association with professionalism in nursing were extracted. These factors were categorized into sociodemographic characteristics, profession-related characteristics, and organizational factors. However, none of the factors had significant associations in the main meta-analysis.

### Sociodemographic characteristics

One of the sociodemographic characteristics is the sex of the participants. In this systematic review, three studies reported that the odds of having a high level of professionalism were higher among female nurses than male nurses (7, 16, 19). Another factor identified was age. Two studies reported a significant association between age and professionalism, with one study indicating that professionalism increased as nurses’ age increased by 1 year (24). However, another study reported that the odds of professionalism decreased when the nurses’ age increased (20). Two other studies reported that marital status was significantly associated with the level of professionalism. Being single (7) and being married (21) in marital status increase the level of professionalism compared to the other marital statuses. The odds of professionalism were higher among nurses with a degree or higher education than nurses with a diploma (7, 23). However, in one study, diploma nurses had a higher level of professionalism than nurses who had a degree or higher education (24). According to the report of one study, the level of professionalism decreased when the monthly salary increased (7).

### Profession-related characteristics

Longer work experience (11, 21, 24), personal satisfaction (17, 21), holding a position (18), membership in a nursing organization (11, 16, 24), collaboration with other healthcare teams (17, 20, 23), training on professionalism (19), a favorable attitude toward the nursing profession (24), strong support from the nursing organization and Ministry of Health (24), and a positive self-image (7, 16) were profession-related characteristics that had a significant association with professionalism in nursing. Another very important factor that had an association with professionalism was job satisfaction, although it is not significant in meta-analysis. According to a report on eight studies (16–23), the odds of having a high level of professionalism were increased when the work experience of nurses was above 5 years^.^

### Organizational factors

Low workload (23), working in other than surgical and ICU wards (17, 24), working in the day shift (11), good organizational culture (7, 16, 17, 22), working environment (24), having a high level of organizational commitment (18), and having life insurance (11) were factors that had an association with professionalism in nursing.

## Discussion

In any profession, including nursing, professionalism is crucial to fulfilling responsibilities effectively. This involves continuous learning, skill development, adherence to professional norms, and a high level of commitment. Improving professionalism among nurses is vital to enhancing healthcare quality. However, there is a lack of comprehensive studies on nursing professionalism in Ethiopia. Therefore, this systematic review aims to provide a thorough understanding of nursing professionalism among Ethiopian nurses.

According to the results, the pooled estimate of the studies on professionalism in nursing in Ethiopia was 54% (95% CI: 42–66%). This finding is in line with a systematic review and meta-analysis performed in Indonesia, which reported that 60% of nurses possess good caring behavior (25), as well as another study in Türkiye (26).

Being female and having a higher educational status were the main factors that had a positive association with professionalism in nursing. As nurses advance in their education, they gain an adequate understanding of the theoretical frameworks and concepts of the nursing profession. This, in turn, improves their intention to love the nursing profession and behave according to its conduct and regulations. This finding is supported by studies conducted in Japan (8), Iran (27), and Türkiye (28).

Several studies have reported that factors such as long years of work experience, membership in a nursing organization, and good cooperation with other healthcare teams are positively associated with professionalism. When nurses have worked in their field for a long period, they have the opportunity to receive ethics and code of conduct training. Additionally, nurses have the chance to care for different types of patients and witness their healing and good prognosis, which may increase their interest in the true essence of the nursing profession. Being members of a nursing organization also allows nurses to meet experienced nurses and share insights from the activities of the nursing organization. Nurses are the heart of hospitals, interacting with different individuals during their time in the hospital. When these interactions are smooth and frequent, they help reduce the tension within the profession. As a result, nurses behave like true nursing professionals and try to improve the quality of the profession. The other very important factor that is associated with professionalism is job satisfaction. Studies show that nurses with favorable job satisfaction are more likely to exhibit a high level of professionalism. This is largely due to increased motivation, high commitment (29), and a greater intention to remain in the profession. Additionally, professionalism and job satisfaction have a bidirectional association. When the level of professionalism is decreased, nurses’ job satisfaction and success are also decreased, and vice versa (30, 31).

Organizational factors are key contributors to professionalism, as reported by different studies. Among these, a low workload is the most significant organizational factor positively associated with professionalism in nursing. The possible justification for this may be that the high workloads lead to fatigue and burnout (26, 32, 33). The other factor is working units, particularly those other than surgical wards and intensive care units (ICUs). Nurses who work in a favorable working environment other than the surgical ward and ICUs are more likely to have a high level of professionalism than nurses working in the surgical ward and ICUs. This may be due to the high workload and stress among nurses working in the ICUs and surgical wards (32). This finding is supported by a study conducted in the United States of America among Korean-American (34), Chinese (35), and Korean (35) nurses.

### Limitations

This systematic review and meta-analysis has its limitations. The primary limitation is the high level of heterogeneity among the included studies, as they use different tools to measure professionalism in nursing. This variability could have an impact on the report.

## Conclusion

The level of professionalism in nursing in Ethiopia is suboptimal. Factors positively associated with professionalism in nursing include being female, having a higher educational level, having long years of experience, having a low workload, having favorable job satisfaction, being a member of a nursing organization, having a good working environment, working in non-stressful units, and having a good organizational culture. Therefore, healthcare professionals, the Ministry of Health, and other stakeholders should focus on interventions to enhance the organizational culture, job satisfaction, working environment, and working schedule for nurses. Moreover, researchers should study the level of professionalism regularly and identify the barriers to improving it.

## Data Availability

The original contributions presented in the study are included in the article/supplementary material, further inquiries can be directed to the corresponding author.
